# An Expanded Role for HLA Genes: *HLA-B* Encodes a microRNA that Regulates IgA and Other Immune Response Transcripts

**DOI:** 10.3389/fimmu.2017.00583

**Published:** 2017-05-19

**Authors:** Nilesh Chitnis, Peter M. Clark, Malek Kamoun, Catherine Stolle, F. Brad Johnson, Dimitri S. Monos

**Affiliations:** ^1^Department of Pathology and Laboratory Medicine, The Children’s Hospital of Philadelphia, Philadelphia, PA, USA; ^2^Department of Pathology and Laboratory Medicine, Perelman School of Medicine, University of Pennsylvania, Philadelphia, PA, USA

**Keywords:** *HLA-B*, miR-6891-5p, mirtron, primary immunodeficiency, selective IgA deficiency, B lymphocyte

## Abstract

We describe a novel functional role for the *HLA-B* locus mediated by its intron-encoded microRNA (miRNA), miR-6891-5p. We show that *in vitro* inhibition of miR-6891-5p impacts the expression of nearly 200 transcripts within the B-lymphoblastoid cell line (B-LCL) COX, affecting a large number of metabolic pathways, including various immune response networks. The top affected transcripts following miR-6891-5p inhibition are those encoding the heavy chain of IgA. We identified a conserved miR-6891-5p target site on the 3′UTR of both immunoglobulin heavy chain alpha 1 and 2 (*IGHA1* and *IGHA2*) transcripts and demonstrated that this miRNA modulates the expression of *IGHA1* and *IGHA2*. B-LCLs from IgA-deficient patients expressed significantly elevated levels of miR-6891-5p when compared with unaffected family members. Upon inhibition of miR-6891-5p, IgA mRNA expression levels were increased, and IgA secretion was restored in the B-LCL of an IgA-deficient patient. These findings indicate that miR-6891-5p regulates *IGHA1* and *IGHA2* gene expression at the posttranscriptional level and suggest that increase in miR-6891-5p levels may contribute to the etiology of selective IgA deficiency.

## Introduction

The major histocompatibility complex (MHC), a 4Mb region on chromosome 6, encompasses over 180 protein coding genes, including numerous genes involved in innate and adaptive immunity (1, 2). This region has been shown to harbor the highest number of disease-associated genetic variants when compared with any other region of comparable size in the human genome (3). Many of these associations lie within the highly polymorphic human leukocyte antigen (HLA) genes (4, 5). Given that 90% of causal autoimmune disease variants are located within non-coding regions of the genome (6), the non-coding regions of HLA genes may also harbor genomic elements that play a functional role in disease pathogenesis. A search for functional genomic elements within the non-coding regions of HLA genes revealed an annotated microRNA (miRNA), hsa-miR-6891 (miR-6891), which is encoded by intron 4 of *HLA-B* (7).

**Figure 1:**
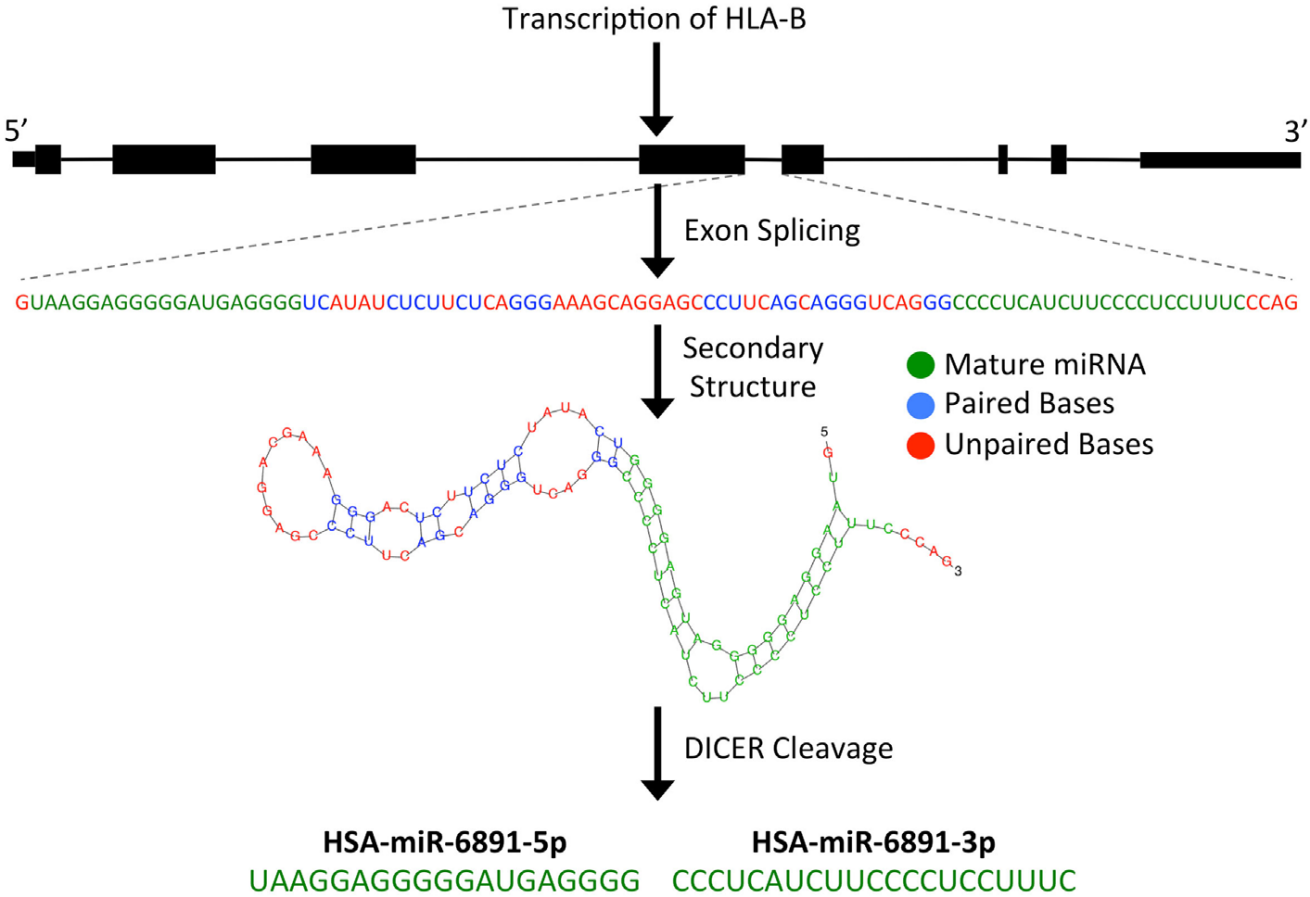
**Predicted biogenesis of HSA-miR-6891**. miR-6891 is derived from intron 4 of *HLA-B*, which upon exon splicing of the *HLA-B* transcript forms a stable pre-miRNA hairpin structure. The pre-miRNA is then processed by the Dicer enzyme to form two mature miRNA products, HSA-miR-6891-5p and HSA-miR-6891-3p.

microRNAs are short (~22 bp), single-stranded, non-coding RNA (ncRNA) transcripts that have been shown to modulate numerous biological processes by regulating the expression of targeted mRNA transcripts through sequence-specific miRNA/mRNA interactions, resulting in the degradation or translational suppression of the targeted mRNA transcript (8). Primary miRNA transcripts are generated by RNA polymerase II or III and form precursor miRNA (pre-miRNA) hairpin structures following processing by the Drosha/DGCR8 microprocessor complex (9). Alternatively, as is the case with miR-6891, a pre-miRNA hairpin may also be formed independently of the Drosha/DGCR8 microprocessor complex. In these instances, a pre-miRNA is formed from an intronic sequence of a gene following exon splicing of the primary mRNA transcript. Given their biogenesis, such miRNA are termed “mirtrons” and are abundant throughout the genome (7, 10). As with other mirtrons, the annotated pre-miRNA hairpin of miR-6891 is believed to be formed from intron 4 of *HLA-B* following splicing of the primary *HLA-B* mRNA transcript and is further processed by the Dicer enzyme to produce two mature, single-stranded miRNA transcripts, miR-6891-5p and miR-6891-3p (7) (Figure 1). Mature miRNAs bind to mRNA transcripts forming a heteroduplex that is loaded onto the RNA-induced silencing complex, resulting in posttranscriptional degradation of the targeted mRNA transcript (11).

The *HLA-B*-encoded miRNA, miR-6891-5p was initially characterized from a meta-analysis of RNA-seq datasets, with reads from both arms of the hairpin (5′ and 3′ arms together) mapping uniquely to the annotated locus within intron 4 of the *HLA-B* gene (7). There is currently no known function of miR-6891-5p. In our current work, we study the physiological role of miR-6891-5p within B lymphocytes through miR-6891-5p inhibition and transcriptome wide mRNA profiling to identify affected transcripts. Our results indicate that miR-6891-5p regulates the expression of numerous transcripts including immunoglobulin heavy chain alpha 1 and 2 (*IGHA1* and *IGHA2*), which was found to be among the most enriched mRNA targets of miR-6891-5p. A binding site of miR-6891-5p that is conserved on the 3′UTR of both *IGHA1* and *IGHA2* was identified by molecular modeling of the two transcripts (*IGHA1* and *IGHA2* have identical 3′UTR sequences) and experimentally validated using a luciferase reporter assay. Additional expression profiling of miR-6891-5p and both *IGHA1* and *IGHA2* transcripts within a cohort of B-lymphoblastoid cell lines (B-LCLs) obtained from patients with selective IgA deficiency and unaffected family members reveals a significant increase in miR-6891-5p expression and an attenuation of *IGHA1* and *IGHA2* expression among affected individuals. Furthermore, inhibition of miR-6891-5p within B-LCLs originating from an IgA-deficient patient resulted in significantly increased expression of *IGHA1* and *IGHA2* mRNA and a significant increase in the amount of secreted IgA. Our findings indicate a novel physiological role of the *HLA-B* gene that extends beyond the antigen-specific immune responses for which it is well known and raises the possibility that the *HLA-B*-encoded miRNA, miR-6891-5p, plays an important role in controlling the expression of many immunologically relevant transcripts.

## Materials and Methods

### HSA-miR-6891 IsomiR Characterization and Sequence Conservation

Full-length annotated *HLA-B* allele sequences were obtained from ImMunoGeneTics (IMGT, version 3.23.0) and aligned using Clustal Omega (12). The multiple sequence alignment was subsequently used to characterize the sequence variability within intron 4 across *HLA-B* alleles. Sequence logo plots for regions encoding the two mature miRNAs, HSA-miR-6891-5p and HSA-miR-6891-3p, were generated using MATLAB (R2014b) in order to visualize sequence variability within the mature miRNA products. Sequence conservation of the pre-miRNA (HSA-miR-6891) hairpin was determined using BLAST (blastn 2.4.0) against the reference genomic sequence database (refseq_genomic) with the following parameter settings: word size of 28, expected value of 10, hitlist size of 100, match/mismatch scores of 1/−2, gapcosts of 0, low complexity filter on, filter string set to L;m, and genetic code set to 1.

### Cell Culture

COX cells (13) were obtained from the International Histocompatibility Working Group, Seattle, WA, USA [(IHW09022) http://www.ihwg.org/hla/index.html]. PGF cells (13) were obtained from the Coriell Biorepository (Cat #GM03107). Cells were culured in RPMI-1640 medium with 15% FBS (Sigma Cat #F2442-500ML). HEK 293T cells (a gift from Xianxin Hua in the Department of Cancer Biology at the University of Pennsylvania, Perelman School of Medicine) were cultured in DMEM (Cat #10-013-CV) media with 10% FBS. Primary B-cells were purified from peripheral blood using EasySep™ Direct Human B Cell Isolation Kit from Stemcells Technologies Cat #19674 and directly used for RNA purification. Selective IgA-deficiency patient cell lines (ID 000018, ID000036, ID000038, and ID000057) and cell lines from unaffected, related family members (ID000037 and ID000058) were originally collected and characterized as part of an initiative by the US Immunodeficiency Network and purchased from the Coriell Biorepository.

### HSA-miR-6891-5p Inhibition

An “inhibition” lentivirus was generated in cultured HEK 293T cells by transfecting with a pEZX-am03 vector (Genecoepia) containing an HSA-miR-6891-5p antisense insert under the control of a CMV promoter. A lentivirus containing similar insert but with a “scrambled” sequence (i.e., random sequence changes all the bases in the seed region) was similarly generated in HEK 293T cells. Media was discarded after 24 h posttransfection, and packaging media was added to the plate. Scrambled and HSA-miR-6891-5p knockdown viruses were collected every 24 h for 2 days.

For transduction, 1.5 × 10^5^ COX cells were plated in 6-well plates, and 2 mL of fresh scrambled or miR-6891-5p knockdown lentivirus was added along with 4 mg/mL polybrene. The plate was centrifuged at 2,500 rpm for 90 min. After 10 h, 2 mL of additional virus with polybrene was added, and the plate was centrifuged at 2,500 rpm for 90 min. After 16 h, 2 mL of media was discarded, and 2 mL of fresh virus and polybrene were added, and the plate was centrifuged at 2,500 rpm for 90 min. Transduction was allowed to continue for an additional 24 h before cells were collected for RNA extraction. RNA was purified using the miRNeasy kit (Qiagen).

### Microarray Analysis

Total RNA extracted from each of the biological replicates of transfected COX cells for both conditions (i.e., inhibition and scrambled control) was used to generate sense-strand cDNA using the *Ambion*^®^ *WT Expression Kit for Affymetrix*^®^ *GeneChip*^®^ *Whole Transcript Expression Arrays* (P/N 4425209). From each of these reactions, 5.5 μg of sense-strand cDNA was fragmented and labeled using the Affymetrix GeneChip WT Terminal Labeling and Hybridization Kit (PN 702880). Fragmented and labeled sense-strand cDNA (3.25 μg) was hybridized to an Affymetrix Human Gene 2.0ST Array. Arrays were washed on an Affymetrix GeneChip Fluidics Station 450 using fluidics protocol FS450_0002 and scanned on Affymetrix GeneChip Scanner 3000.

Raw data (CEL) files were imported and processed within MATLAB (R2014b). Raw data were first background adjusted using the robust multiarray average procedure, followed by quantile normalization with median polishing and probe level summarization using a custom CDF annotation file (14–18). Those probes on the array with null values for at least one sample were removed so as not to confound subsequent analysis. Principal component analysis (PCA) of the normalized dataset was performed within MATLAB (R2014b) in order to visualize sample clustering and identify sample outliers (19). Differentially expressed transcripts were identified between the miR-6891-5p inhibition samples and control samples (scrambled vector) using significant analysis of microarrays (20). Differentially expressed transcripts were identified using a false discovery rate (FDR) cutoff of 0.05 (21, 22) and a fold change cutoff of 2. Hierarchical clustering of differentially expressed transcripts for each replicate was performed using MATLAB (R2014b). Functional enrichment was performed using DAVID (23). Significant gene ontology (GO) biological processes (BP_FAT) and molecular functions (MF FAT) were determined using a *p*-value cutoff of 0.05. Raw microarray data are publicly available and may be accessed via NCBI GEO (link provided upon acceptance).

### Computational Prediction of HSA-miR-6891-5p Targets

Computationally predicted mRNA targets of HSA-miR-6891-5p were identified throughout the entirety of every annotated gene using miRWalk2.0 with default parameters and every available database, including miRWalk, miRDB, PITA, MicroT4, miRMap, RNA22, miRanda, miRNAMap, RNAhybrid, miRBridge, PICTAR2, and Targetscan (24). The set of genes with a computationally predicted miRNA binding site for miR-6891-5p were then intersected with the set of targets identified by microarray analysis.

### HLA Genotyping

Genomic DNA was extracted from the IgA-deficient B-LCLs using the Qiagen Gentra Puregene Blood Kit (Cat No./ID: 158389). Sequencing libraries were generated for each sample using the Omixon Holotype HLA Genotyping Kit as previously described (25). The library was then denatured with NaOH and diluted to a final concentration of 8 pM for optimal cluster density and 600 µL was loaded into the MiSeq reagent cartridge (version 2 500 cycle kit). Samples were demultiplexed on the instrument, and the resulting FASTQ files were used for further analysis. All samples were genotyped at the *HLA-B* locus using Omixon Target (version 1.8). High-resolution *HLA-B* genotyping results are found in Table S4 in Supplementary Material.

### Quantitative PCR

Total RNA was extracted from cells using a Qiagen miRNeasy kit (Cat #217084) per manufacturer’s protocol (26). Total RNA was reverse transcribed using the Qiagen miRNA Reverse Transcription kit (Cat #218160). qPCR was performed on cDNA generated by reverse transcription using a miSCRIPT SYBR Green PCR kit (Cat #21803). Primers for HSA-miR-6891-5p were obtained from Qiagen (Cat #MS00048202). Primers for *IGHA1, IGHA2*, β*-actin*, and *HLA-B* (sequences provided below) were obtained from IDT. Selective IgA patient cells were HLA genotyped, and qPCR primers were designed to amplify all genotyped *HLA-B* mRNA transcripts. Selective IgA deficiency cells were harvested, and total RNA was purified using Qiagen miRneasy kit. This RNA was used for qPCR using *HLA-B* and miR-6891-5p primers. Data were normalized to actin. Significance was assessed using an unpaired one-tailed *t*-test.

Primer sequences:

(i) IGHA1
  - Forward 5′-TTCCCTCAACTCCACCTACC-3′
  - Reverse 5′-CGTGAGGTTCGCTTCTGAAC-3′
(ii) IGHA2
  - Forward 5′-GAGACCTTCACCTGCACTG-3′
  - Reverse 5′-TGTGTTTCCGGATTTTGTGATGT-3′
(iii) β-actin
  - Forward 5′-AGAGCTACGAGCTGCCTGAC-3′
  - Reverse 5′-AGCACTGTGTTGGCGTACAG-3′
(iv) *HSA*-miR-6891-5p mCherry Reporter
  - Forward 5′-CAGACCGCCAAGCTGAA-3′
  - Reverse 5′-GAGCCGTACATGAACTGAGG-3′
(v) *HLA-B* mRNA
  - Forward 5′-GTCCTAGCAGTTGTGGTCATC-3′
  - Reverse 5′-CAAGCTGTGAGAGACACATCAGA-3′

### IgA ELISA

COX and PGF cells were cultured in RPMI-1640 media. After 72 and/or 120 h, media were collected, and IgA secretion was analyzed using Ready-SET-Go ELISA kit (27, 28) (Cat #88-50600) from Affymetrix (CA) per manufacturer’s protocol. Significance was assessed using the one-tailed *t*-test.

### Luciferase Assay

The complete (48 nucleotide) 3′UTR sequence of the *IGHA1* gene (which is identical to the 3′UTR sequence of the *IGHA2* gene), containing the HSA-miR-6891-5p binding site, was synthesized with *Pme*I and *Xba*I sites on either end (IDT) and gel purified using a QIAquick Gel Extraction Kit (Qiagen Cat #28704). The product was ligated into the pmiRGLO plasmid (Promega, WI, USA) digested with *Pme*I and *Xba*I (New England Biolabs, MA, USA) downstream of the PGK promoter and luciferase gene.

For the luciferase assay, 1 × 10^6^ HEK 293T cells were cultured in multiwell plates and, after 24 h, were transfected with either the wild-type *IGHA1* 3′UTR or mutant *IGHA1* 3′UTR construct using Fugene 6 (Promega, Cat #E2691). Some of these cells were also transfected with either HSA-miR-6891-5p antisense or overexpression constructs. After 24 h, the cells were assayed for luciferase activity using the Dual-Luciferase^®^ Reporter Assay System (Promega, Cat #E1910) (29). For each measurement, firefly luciferase data were normalized to renilla luciferase. Significance was assessed using Student’s *t*-test.

## Results

### miR-6891 Sequence Variability

**Figure 2:**
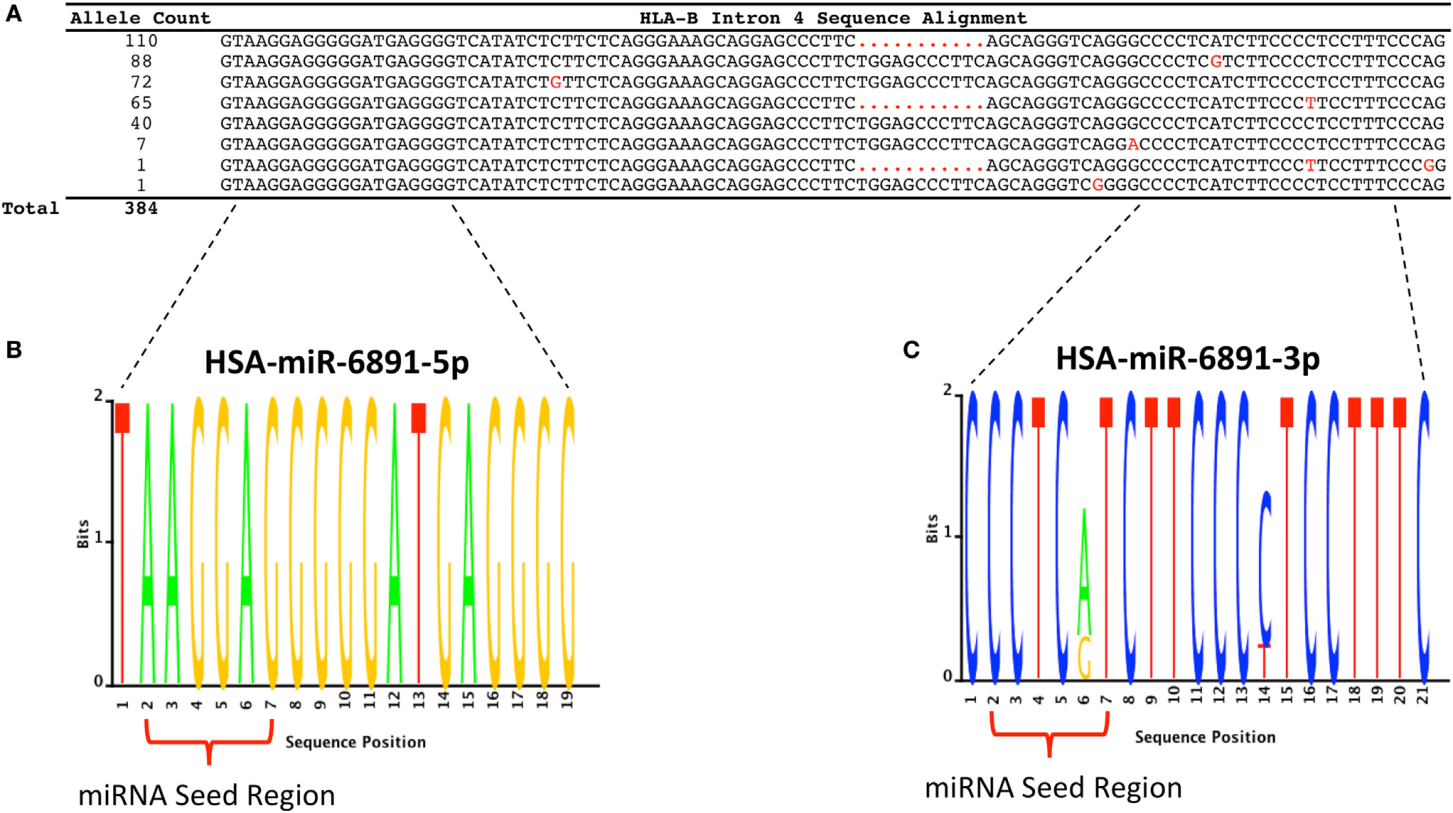
***HLA-B* intron 4 sequence variability and miR-6891 isomiR characterization**. **(A)** There are 384 annotated *HLA-B* alleles with full-length sequence annotation within the ImMunoGeneTics (IMGT) database (release 3.25), with each allele represented by one of eight unique intron 4 sequence motifs. The aligned sequence motifs are provided along with their allele frequency within IMGT and polymorphic positions (highlighted in red). **(B)** Sequence logo plot depicting the lack of polymorphism within HSA-miR-6891-5p. **(C)** Sequence logo plot depicting polymorphic sites within HSA-miR-6891-3p at positions 6 and 14 of the mature miRNA.

Following transcript splicing, intron 4 of *HLA-B* is predicted to form a pre-miRNA hairpin that is further processed by the Dicer enzyme into two mature miRNA products, miR-6891-5p and miR-6891-3p (7) (Figure 1). Given the highly polymorphic nature of the *HLA-B* locus, we explored miR-6891 sequence variants (isomiRs) by interrogating the sequences of intron 4 among the 384 full-length annotated *HLA-B* alleles in the international IMGT database (IMGT/HLA, release 3.25) (30). Among those, only eight unique sequence motifs were observed (Figure 2A). Remarkably and despite the very polymorphic nature of the *HLA-B* gene, there is no sequence variation within miR-6891-5p (Figure 2B) and only two polymorphic sites within the mature miR-6891-3p arm, occurring at positions 6 and 14 of the mature miRNA (Figure 2C). Each of these intronic sequences form stable pre-miRNA hairpin structures with secondary structure minimum free energy values ranging from −43 to −54 kcal/mol. We selected miR-6891-5p for additional study because its conserved sequence suggests an important biological role. The pre-miRNA hairpin sequence of hsa-miR-6891 is evolutionarily conserved, with 90% sequence identity among six primate species including *Homo sapiens, Gorilla gorilla, Nomascus leucogenys, Chlorocebus sabaeus, Macaca nemestrina*, and *Macaca mulatta*. In contrast, the closest homolog of hsa-miR-6891 within the mouse genome, which lies within intron 5 of the H2-T10 gene, has only 48% (45/93 base positions identical) sequence conservation with hsa-miR-6891, and there is no annotated miRNA encoded within this locus (miRbase release 21).

### miR-6891-5p Targeting in B-Lymphocytes

To study the function of miR-6891-5p, an appropriate *in vitro* cell model was first identified by examining the expression level of miR-6891-5p within two B-LCLs, PGF and COX, as well as immortalized HEK293T cells and primary B-lymphocytes (Figure S1 in Supplementary Material). Our qPCR results indicate that miR-6891-5p is expressed in every cell type analyzed, with B-LCLs exhibiting the highest and most uniform expression of miR-6891-5p across biological replicates. For this reason, B-LCLs (COX cells) were selected as our model system to further study the role of miR-6891-5p.

**Figure 3:**
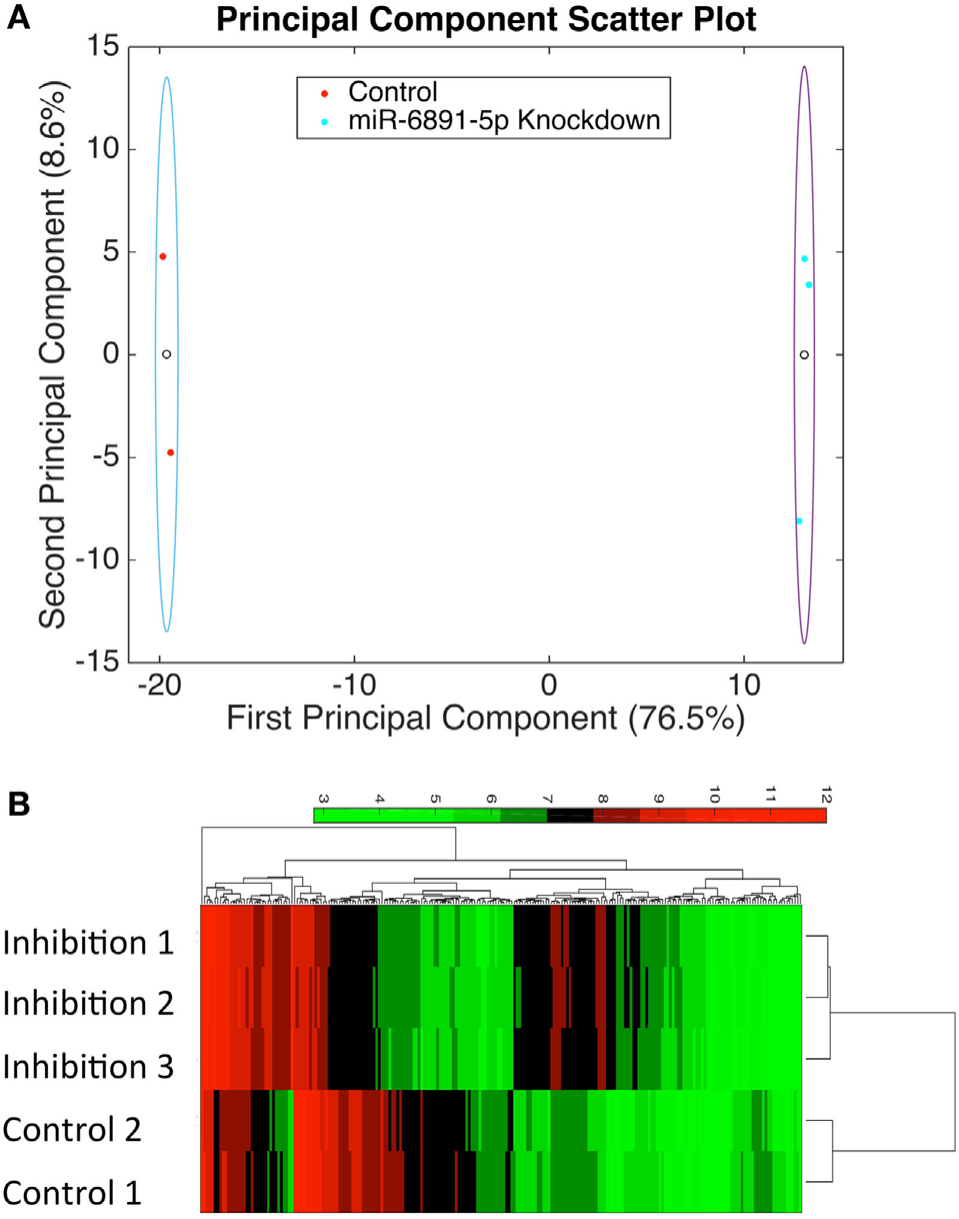
**Identification of potential miR-6891-5p targets**. COX cells were transduced with lentiviruses expressing either antisense of HSA-miR-6891-5p or scrambled control, and altered mRNA transcript levels were assessed using microarrays. **(A)** Principal component analysis (PCA) was performed in order to visualize sample clustering and assess the variation among biological replicates (*N* = 3 experimental and 2 controls samples). Clear circles represent the centroid of the sample clusters, and the ellipse represents 2× the standard deviation in the *x*- and *y*-axes, respectively. The first principal component accounts for 76.5% of the variance within the dataset, while the second principal component accounts for 8.6% of the variance within the dataset. **(B)** Hierarchical clustering of samples based upon identified differentially expressed transcripts from microarray analysis.

To identify putative target transcripts of miR-6891-5p, we transduced COX cells with a lentiviral construct expressing the antisense transcript of miR-6891-5p to inhibit the activity of miR-6891-5p. Upon transduction, transcripts targeted by miR-6891-5p are expected to be more abundant within the transduced cells since miR-dependent degradation has been inhibited. The experimental design included COX cells transduced with either the lentiviral construct expressing the antisense sequence (inhibition) or scrambled antisense sequence (control) of miR-6891-5p. Adequate and comparable expression of lentiviral constructs from both experimental conditions was observed (Figures S2 and S3 in Supplementary Material). Affymetrix Human Gene 2.0ST Arrays were used to assess transcript expression levels between the miR-6891-5p inhibition and control sample groups. PCA (Figure 3A) of the normalized microarray data demonstrates excellent clustering of the two distinct cell populations, indicating distinct and reproducible mRNA expression profiles among biological replicates. Transcripts with significant differential expression between the miR-6891-5p inhibition and control sample groups were identified. One hundred four upregulated and 99 downregulated transcripts were observed within the miR-6891-5p inhibition sample group when compared with the control group, using a fold change cutoff of ≥2 and a FDR cutoff of 0.05 (Figure 3B; Tables S1 and S2 in Supplementary Material, respectively). Since miRNA are known to bind and downregulate the expression of targeted mRNA transcripts, only those transcripts that were identified as upregulated in the miR-6891-5p inhibition sample group were considered to be putative direct targets of miR-6891-5p (Table S1 in Supplementary Material), whereas the set of downregulated transcripts may be related to indirect effects of miR-6891-5p inhibition (Table S2 in Supplementary Material). The potential binding sites of miR-6891-5p within the 104 upregulated transcripts were identified using an *in silico* miRNA target prediction algorithm. Among the 104 empirically identified putative targets of miR-6891-5p, 61 (~58%) were found to harbor a computationally predicted miRNA binding site for miR-6891-5p (Table S1 in Supplementary Material).

Functional analysis of differentially expressed transcripts was performed by determining the enriched GO biological processes of upregulated and downregulated transcripts (Table S3 in Supplementary Material). Significantly upregulated transcripts were found to be involved in numerous immunological processes including leukocyte and mast cell activation (*GIMAP5, EGR1, NDRG1*, and *LCP2)* and various cellular processes including T-cell antigen receptor-mediated signaling (*LCP2*) and T-cell quiescence (*GIMAP5*). Also, the significant upregulated transcripts are 11 DNA-binding proteins and transcription factors (*FOS, EGR1, LEF1, TP63, HIST1H2AG, ZFHX4, ZNF730*, and *ZNF83*) including the transcriptional repressor genes SNAI2, PCGF2, and ZNF253. Significantly downregulated transcripts, following miR-6891-5p inhibition, are involved in numerous immunological processes including cytokine production (*FCER1G, HMOX1, IFNG, IL10, NFATC2, SIRT1*, and *TSPAN6*), regulation of B-cell-mediated immunity (*FCER1G, IFNG*, and *IL10*), inflammation (*CCR1, CXCL10, FCER1G, HMOX1, IL10, PNMA1*, and *PPARG*), and the immunoglobulin-mediated immune response (*FCER1G, IFNG*, and *IL10*).

### miR-6891-5p-Mediated Regulation of IgA

**Figure 4:**
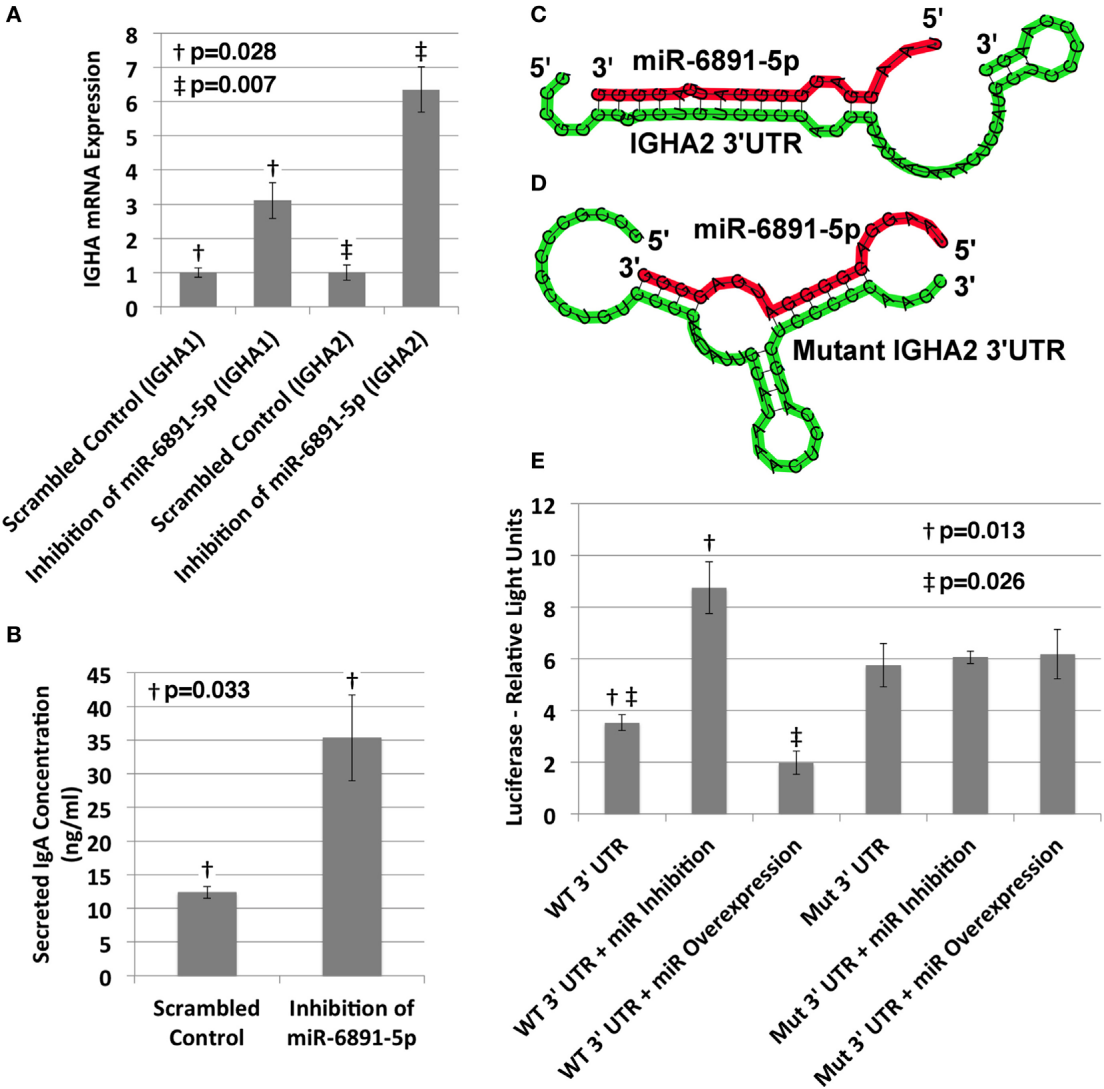
**Validation of miR-6891-5p-mediated posttranscriptional regulation of immunoglobulin heavy chain alpha 1 and 2 (*IGHA1* and *IGHA2*) transcripts**. **(A)** COX cells were transduced with lentiviral constructs expressing either the scrambled control or antisense sequence of miR-6891-5p. Cells were harvested after 48 h of transduction, total RNA was purified, and both *IGHA1* and *IGHA2* expression were analyzed by qPCR (ΔΔCt, standard error bars shown, *n* = 3). **(B)** COX cells (5 × 10^8^) were transduced with lentiviral construct expressing either the scrambled control or antisense sequence of miR-6891-5p. After 120 h, media were collected and analyzed by ELISA using IgA antibody (standard error bars shown, *n* = 3). **(C)** Predicted binding site and heteroduplex formed between the wild-type (WT) 3′UTR of *IGHA2* and miR-6891-5p. The heteroduplex formed with *IGHA1* is identical to that shown. **(D)** Predicted binding site and heteroduplex formed between the mutated (Mut) 3′UTR sequence of *IGHA2* and miR-6891-5p. **(E)** Either the wild-type (WT) or mutated (Mut) 3′UTR sequence of *IGHA2* was cloned downstream of the luciferase reporter, creating two separate constructs. The wild-type or mutant luciferase constructs alone or together with either the miR-6891-5p expression construct (miR overexpression) or the antisense miR-6891-5p expression construct (miR inhibition) were transfected into HEK293T cells. Luciferase assay was performed 24 h after transfection (standard error bars shown, *n* = 3). All *p*-values shown are calculated using a *t*-test.

The IgA heavy chain encoding transcript was among the most significantly upregulated transcripts following inhibition of miR-6891-5p identified from the microarray analysis (8.5-fold change, FDR = 0.02). To study the role of miR-6891-5p on the abundance of both the IgA mRNA transcript and secreted IgA protein, IgA secreting COX cells were transduced with a lentivirus expressing either the antisense of miR-6891-5p (miR-6891-5p inhibition) or a scrambled antisense sequence of miR-6891-5p (control). Inhibition of miR-6891-5p within COX cells significantly increased the abundance of both the *IGHA1* and *IGHA2* mRNA transcripts (*p* = 0.028 and *p* = 0.007, respectively) (Figure 4A) and secreted IgA protein (*p* = 0.033) (Figure 4B) compared to cells transduced with the scrambled control. These findings demonstrate that miR-6891-5p inhibits the expression of both *IGHA1* and *IGHA2*. Our *in silico* molecular modeling of both *IGHA1* and *IGHA2* transcripts reveals an energetically favorable binding site of miR-6891-5p on the 3′UTR of *IGHA1* that is 100% conserved within the 3′UTR of the *IGHA2* transcript, suggesting that miR-6891-5p may bind and regulate the expression of both transcripts. The identified non-canonical heteroduplex contains limited base pairing between the miRNA seed region (positions 2–7 of the 5′ end) and the conserved 3′UTR sequence of the *IGHA1* and *IGHA2* transcripts (Figure 4C).

To validate functional targeting of the modeled miR-6891-5p-binding site within the conserved 3′UTR sequences of both *IGHA1* and *IGHA2*, the UTR sequence was fused to a plasmid-based luciferase reporter and transfected into HEK293T cells. HEK293T cells express miR-6891-5p but not IgA and thus provide a cell model system to study *IGHA* 3′UTR targeting without competitive binding from endogenously expressed *IGHA* mRNA. These cells were also transfected with either the miR-6891-5p antisense expression plasmid to inhibit endogenously expressed miR-6891-5p (miR inhibition) or a plasmid expressing miR-6891-5p to increase the level of the endogenously expressed miR-6891-5p (miR overexpression). Inhibition of miR-6891-5p significantly increased luciferase activity (*p* = 0.013), whereas overexpression of miR-6891-5p significantly attenuated luciferase activity (*p* = 0.026). Further validation of the binding site was performed by mutating the 3′UTR sequence underlying the binding site of miR-6891-5p (Figure 4D) and fusing it to a plasmid-based luciferase reporter, which was then transfected into HEK293T cells. These cells were also transfected with either the miR-6891-5p antisense expression plasmid to inhibit endogenously expressed miR-6891-5p or a plasmid expressing miR-6891-5p to increase the level of the endogenously expressed miR-6891-5p. In contrast to the wild-type 3′UTR luciferase experiments, no modulation of miR-6891-5p (inhibition or overexpression) was able to affect luciferase activity (Figure 4E), indicating that miR-6891-5p was unable to bind the mutant 3′UTR sequence. Together, these results suggest direct miR-6891-5p targeting on the 3′UTR of both the *IGHA1* and *IGHA2* transcripts.

### Implications for Selective IgA Deficiency

**Figure 5:**
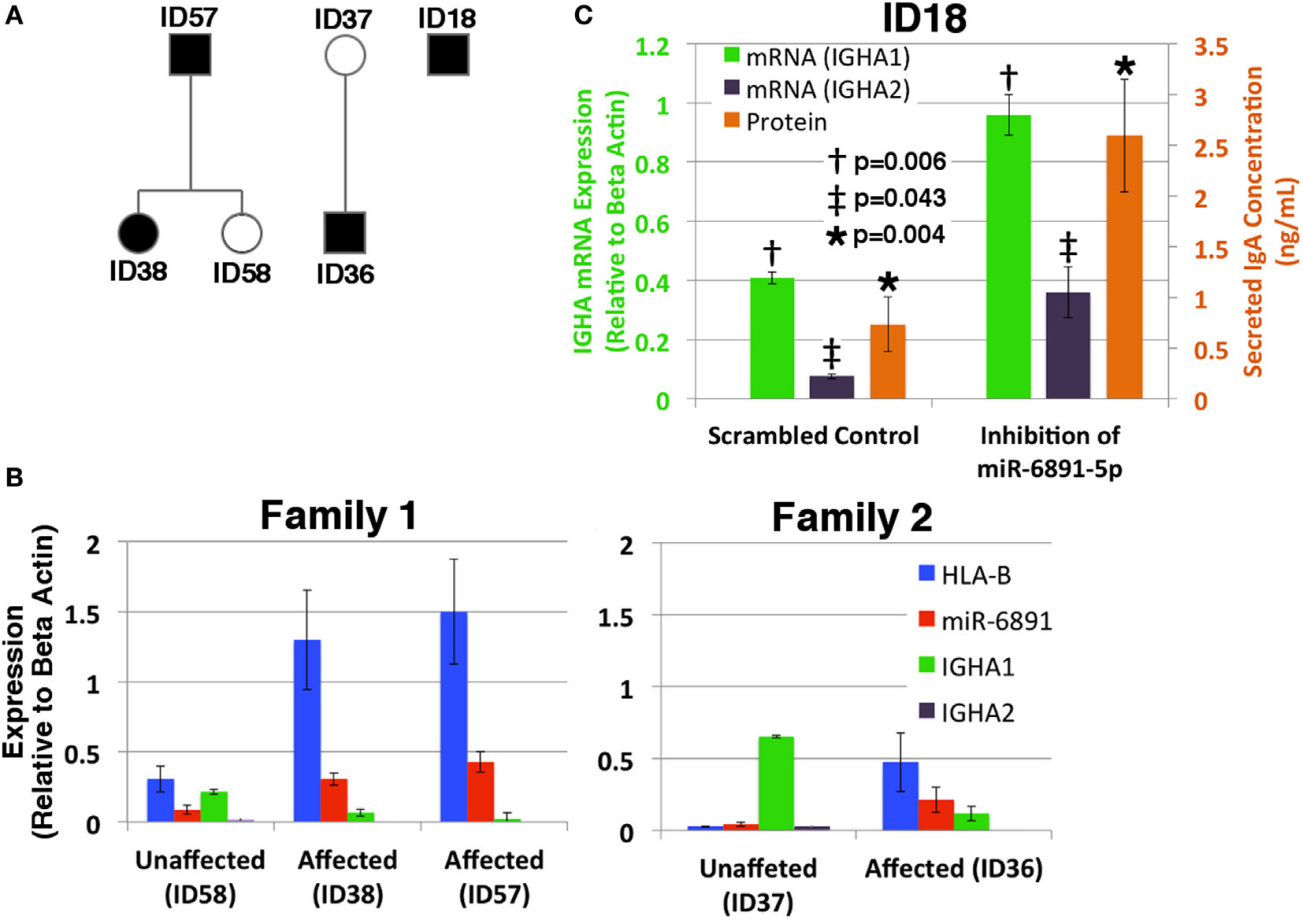
**Exploring the role of miR-6891-5p in selective IgA deficiency (A) pedigree of affected (proband, black shadowing) and unaffected (white shadowing) family members is presented in panels B and C**. **(B)** *HLA-B*, miR-6891-5p *IGHA1*, and *IGHA2* expression (qPCR) among IgA-deficient B-LCLs collected from affected individuals and unaffected family members (standard error bars shown, *n* = 3). **(C)** Selective IgA deficient cell line ID18 was transduced with a lentiviral construct expressing either the antisense miR-6891-5p (miR-6891-5p inhibition) or the scrambled sequence of antisense miR-6891-5p (control). Total RNA was purified, and *IGHA1 and IGHA2* mRNA transcript levels were analyzed by qPCR (*y*-axis shown on left of plot, standard error bars shown, *n* = 3). After 24 h, media was collected and analyzed by ELISA using anti-IgA antibody (*y*-axis shown on right of plot, standard error bars shown, *n* = 3). All *p*-values shown are calculated using a *t*-test.

Given our findings, we investigated the putative role of miR-6891-5p on the expression and secretion of IgA within B-LCLs obtained from two familial cohorts, consisting of individuals affected by selective IgA deficiency and unaffected relatives (Figure 5A). In order to design effective qPCR primers that amplify the *HLA-B* mRNA transcripts of each individual, high-resolution HLA genotyping was performed on all affected and unaffected individuals for eight HLA loci (Table S4 in Supplementary Material). Phased MHC haplotypes were inferred from related individuals using the family pedigree when available (ID57, ID58, ID38, ID37, and ID36) or from common MHC haplotypes otherwise (ID18). Expression of *HLA-B*, miR-6891-5p, *IGHA1*, and *IGHA2* was quantified by sequence-specific qPCR primers (Figure 5B). We observe that *IGHA1* is the primarily expressed heavy chain transcript of IgA across all individuals and demonstrate an inverse correlation between miR-6891-5p expression and *IGHA1* expression (Pearson correlation −0.87), as well as a strong correlation between *HLA-B* and miR-6891-5p expression (Pearson correlation 0.96), across all patient samples. Both families showed increased expression of both *HLA-B* (ID36/ID37 = 18.6×; ID38/ID58 = 4.2×; ID57/ID58 = 4.9×) and miR-6891-5p (ID36/ID37 = 5.3×; ID38/ID58 = 3.5×; ID57/ID58 = 16.8×). In all cases, miR-6891-5p expression was found to be less than that of the host gene, *HLA-B*. Additionally, inhibition of miR-6891-5p, in an IgA-deficient cell line (ID18), led to a significant increase (~3×) in both *IGHA1* and *IGHA2* transcript abundance (*p* = 0.006 and *p* = 0.043, respectively) as well as a significant increase in the concentration of secreted IgA protein (*p* = 0.004) (Figure 5C).

## Discussion

Human leukocyte antigen molecules are best known for their role in the antigen-specific immune response and in differentiating self from non-self. However, our research suggests a novel regulatory role of the *HLA-B* gene mediated by a cotranscribed miRNA, miR-6891-5p, encoded within intron 4 of the *HLA-B* transcript (7). Our analysis reveals that miR-6891-5p is 100% conserved across every annotated full-length *HLA-B* allele, while miR-6891-3p contains two polymorphic locations, including one within the seed region. Our previous research quantifying class I HLA allele sequence diversity demonstrates that intron 4 of *HLA-B* is the most conserved intron among class I HLA genes (31). The sequence conservation of miR-6891-5p among *HLA-B* alleles, as well as among other non-human primates suggests that this miRNA plays an important regulatory role and forms the basis for our functional study of miR-6891-5p.

Our functional study of miR-6891-5p within B-LCLs suggests that miR-6891-5p regulates the expression of nearly 200 transcripts, which are involved in numerous immunological processes. Since miRNAs are known to attenuate the posttranscriptional expression of targeted transcripts, inhibition of miR-6891-5p would be expected to upregulate the expression of directly targeted transcripts. However, because miR-6891-5p inhibition was found to upregulate the expression of several transcription factors (all of which contain a computationally predicted miR-6891-5p-binding site), it is possible that many of the observed differentially expressed transcripts may result from indirect, downstream effects of miR-6891-5p inhibition that are mediated by targeted transcription factors. Because three of the identified targeted transcription factors are known transcriptional repressors (SNAI2, PCGF2, and ZNF253), it is likely that the upregulation of these repressors following miR-6891-5p inhibition would attenuate the transcription of numerous genes, resulting in the observed downregulation of numerous transcripts following miR-6891-5p inhibition. Similarly, the observed upregulation of transcriptional activators (*LEF1, EGR1, TP63*, and *FOS*) following miR-6891-5p inhibition may upregulate the transcription of numerous genes that are not direct targets of miR-6891-5p and may partially explain the observed upregulation of genes that do not harbor a computationally predicted binding site of miR-6891-5p. Together these data suggest that miR-6891-5p not only regulates the posttranscriptional expression of directly targeted transcripts but may also modulate the transcription of numerous other genes indirectly, through miR-6891-5p-mediated translational repression of targeted transcriptional activators and/or repressors. These results suggest an important physiological role of miR-6891-5p within B-LCLs. The ubiquitous expression of HLA-B also suggests that miR-6891-5p may play a broader role in various tissues and cellular phenotypes and is the subject of ongoing research.

Upon miR-6891-5p inhibition, transcripts encoding the heavy chain of IgA were found to be among the top identified upregulated transcripts. This particular target of miR-6891-5p was selected for further validation since immunoglobulin production is a key function of plasma cells and no miRNA has been shown to directly bind and regulate immunoglobulin expression, although miR-155 has been shown to indirectly influence immunoglobulin expression through regulation of B-cell differentiation and maturation (32). Despite the lack of a predicted miR-6891-5p-binding site on either the *IGHA1* or *IGHA2* transcript using current computational miRNA target prediction algorithms (Table S1 in Supplementary Material), our molecular modeling of the miR-6891-5p, *IGHA1*, and *IGHA2* transcripts reveals an energetically favorable, non-canonical heteroduplex formation, with limited base pairing within the miRNA seed region (traditionally defined as base positions 2–7 of the 5′ end of the mature miRNA) and the identified target site on the 3′UTR of both the *IGHA1* and *IGHA2* transcripts. Experimental validation of the modeled miR-6891-5p binding site within the 3′UTR sequence by the luciferase reporter assay (including control experiments using a mutated sequence of the miR-6891-5p binding site) indicates miR-6891-5p-mediated posttranscriptional regulation of IgA through the modeled, non-canonical interaction with the 3′UTR of both the *IGHA1* and *IGHA2* transcripts. Because the binding site of miR-6891-5p on the 3′UTR of both *IGHA1* and *IGHA2* transcripts is 100% conserved, our results indicate that miR-6891-5p regulates the expression of both transcripts through an interaction within a conserved target site present on the 3′UTR of both transcripts, effectively mediating the posttranscriptional expression of both the *IGHA1* and *IGHA2* transcripts. Recent research suggests that the existence of non-canonical heteroduplex formations between a miRNA and its target may be more prevalent than previously thought (33). This in turn may lead to false-negative miRNA target predictions by algorithms that rely on a high degree of Watson-crick base complementary between the seed region of a given miRNA and the predicted target site. Together these considerations suggest that the number of significantly upregulated transcripts following inhibition of miR-6891-5p that harbor a computationally predicted miR-6891-5p-binding site (58%) may be an underestimate of the true number of directly targeted transcripts identified by microarray expression analysis following miR-6891-5p inhibition.

Our initial findings led us to investigate the putative role of miR-6891-5p in the pathophysiology of selective IgA deficiency within B-LCLs obtained from affected individuals and unaffected family members. Selective IgA deficiency is the most common form of primary immunodeficiency and is characterized by the dysregulation of IgA synthesis within immature B lymphocytes resulting in diminished levels of IgA in patient serum (34, 35). B-LCLs obtained from affected individuals were found to express significantly increased levels of both *HLA-B* and miR-6891-5p when compared with unaffected family members. The expression of miR-6891-5p and the host gene, *HLA-B*, was highly correlated (Pearson 0.96). Consistent with our previous findings, expression of miR-6891-5p was inversely correlated with *IGHA1* and *IGHA2* expression (Pearson −0.8 and −0.86, respectively). Abundance of miR-6891-5p was found to be less than that of the host gene, *HLA-B*, which is consistent with previous findings correlating mirtron and host gene expression (10). Inhibition of miR-6891-5p within B-LCLs isolated from a patient with selective IgA deficiency was found to significantly increase the abundance of both *IGHA1* and *IGHA2* mRNAs as well as secreted IgA protein. Although the genetic etiology of the disease remains to be fully elucidated, a recent GWAS study has demonstrated a primary association within the HLA class II region and an independent association within the HLA class I (*HLA-B*) and HLA class III region of the MHC, suggesting a complex genetic association resulting from the combined effects of variants spanning the class I, II, and III HLA regions (36). Additionally, the HLA-A*01-B*08-DRB1*0301-DQB1*02 (DR3), HLA-B*14-DRB1*0102-DQB1*05 (DR1), and HLA-B*44-DRB1*0701-DQB1*02 (DR7) MHC haplotypes have all been associated with IgA deficiency, while the HLA-DRB1*1501-DQB1*06 (DR2) MHC haplotype has been shown to confer protection against IgA deficiency (36, 37). Previous research further demonstrates that the prevalence of IgA deficiency among HLA-B8-DR3 homozygous individuals ranges between 1.7% (38) and ~13% (39, 40). Furthermore, the HLA genotyping of all family members analyzed by our study (affected and unaffected by IgA deficiency) reveals that 3/4 of the affected individuals (ID57, ID38, and ID18) and all (2/2) of the unaffected individuals are heterozygous for the B8-DR3 haplotype (Table S4 in Supplementary Material), further demonstrating that IgA deficiency likely stems from a number of heterogeneous genetic effects acting in a concerted manner (35). Considering these findings along with the absence of polymorphisms within the miR-6891-5p gene and the observed significantly elevated expression of *HLA-B* and miR-6891-5p within B-LCLs from patients with selective IgA deficiency, our data suggest a disease model in which the accumulation of miR-6891-5p transcripts may play a role in the pathophysiology of the disease by attenuating expression of IgA. Although the precise mechanism by which this occurs is the subject of ongoing research, it is possible that the primary GWAS signals previously reported by others may result from polymorphisms within an eQTL or other genomic elements present on the associated susceptible MHC haplotypes that result in the increased expression of miR-6891-5p. Thus, it is possible that altered miR-6891-5p expression may be a contributing factor in the pathophysiology of selective IgA deficiency and warrants further study within primary tissue samples from affected individuals.

Our study is the first to describe a functional role of the HLA-B-encoded miRNA, miR-6891-5p, and signifies a paradigm shift in the fundamental understanding of the role of the HLA-B gene. Our recent efforts to characterize the miRNA transcriptome of BLCLs suggest that other HLA genes also encode functional miRNA transcripts (41). Together these works lay the groundwork for further studies investigating the role of HLA encoded miRNAs in regulating transcripts involved in the immune response and other metabolic processes. Previous research demonstrates that 90% of causal autoimmune disease variants are located within non-coding regions of the genome (6). Given the ubiquitous expression of class I HLA genes within nearly all nucleated cells, detailed characterization of the regulatory role of HLA-encoded miRNAs across various cell types and disease states may reveal interesting new insights offering a potential explanation for some of the reported disease associations within non-coding regions of the MHC. Thus, our current work necessitates additional efforts to better characterize and study the functional role of miRNA transcripts originating from among the most complex and under characterized region of the genome, the MHC.

## Author Contributions

PC, DM, and NC conceived and designed the experiments with input from FBJ, MK, and CS. All laboratory experiments were conducted by NC within the laboratories of DM and FBJ. All computational analysis was performed by PC. PC generated the figures, with input from DM, NC, MK, and FBJ. PC drafted the manuscript with input from DM, FBJ, MK NC, and CS. All authors approved the final manuscript prior to submission.

## Conflict of Interest Statement

The authors declare that the research was conducted in the absence of any commercial or financial relationships that could be construed as a potential conflict of interest.
