# Supplementary material for: An Expanded Role for HLA Genes: HLA-B Encodes a microRNA that Regulates IgA and Other Immune Response Transcripts
Source: Front Immunol. 2017 May 19;8:583. doi: 10.3389/fimmu.2017.00583 (PMC5437213; doi:10.3389/fimmu.2017.00583)
Supplement: Supplementary file 1 [file Data_Sheet_1.pdf]

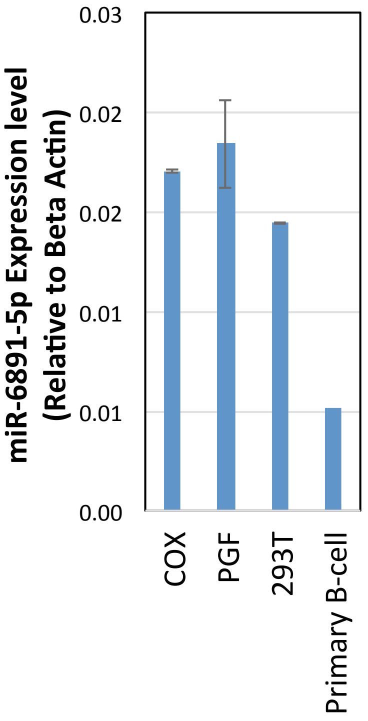

Figure S1. Expression of HSA-miR-6891-5p in cultured COX, PGF and HEK293T cells and primary human B-cells purified from total blood. Q-PCR was performed using HSA-miR-6891-5p specific primers and normalized with β-actin Q-PCR data.

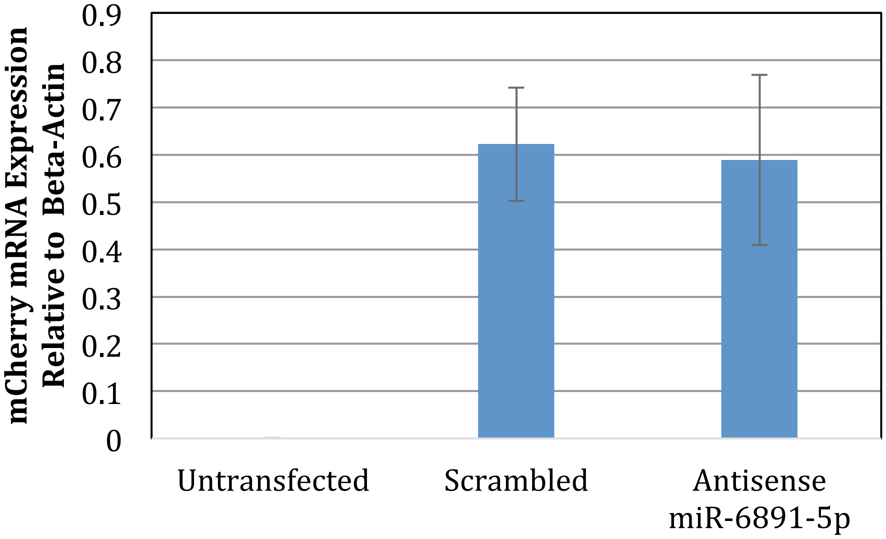

Figure S2. Expression of control (scrambled) and antisense of miR-6891-5p in transduced COX cells. Total RNA was purified and, to confirm the antisense production, the level of mCherry reporter mRNA was analyzed as an indicator of antisense expression. Standard deviation bars show the results of 3 biological replicate experiments. No signal could be detected in untransduced COX cells.

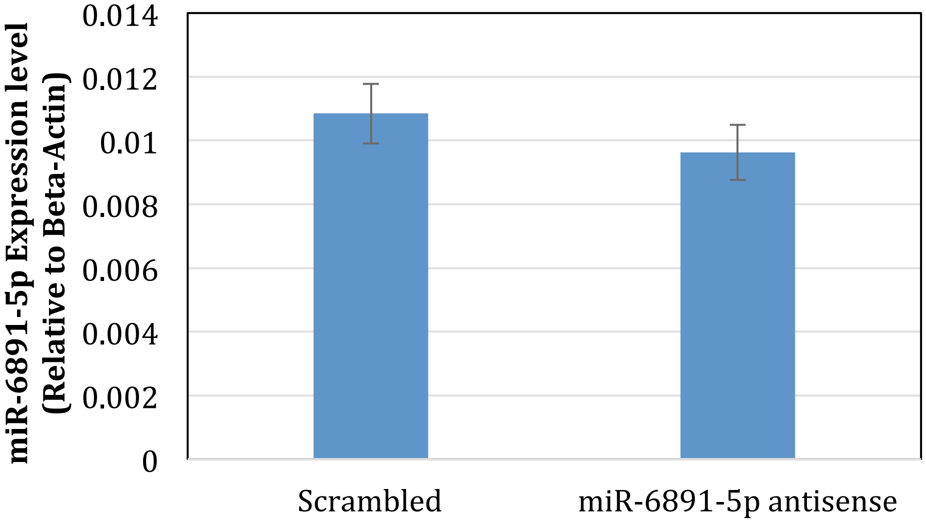

Figure S3. COX cells were transduced with lentiviral constructs expressing either the scrambled control or antisense sequences of miR-6891-5p. Total RNA was purified and miR-6891-5p expression levels were analyzed by Q-PCR in order to demonstrate that miR-6891-5p expression is comparable between the two conditions and unaffected by transduction. Standard deviation shows results of 3 biological replicate experiments.

Ensemble Gene ID	Gene Symbol	Fold Change	FDR
ENSG00000226777	KIAA0125	22.7	1.2E-02
ENSG00000211890	IGHA2	8.5	2.0E-02
ENSG00000186522	SEPT10	7.8	3.8E-03
ENSG00000229807	XIST	7.5	2.0E-03
ENSG00000133124	IRS4	6.4	4.5E-03
ENSG00000237438	CECR7	6.3	2.4E-02
ENSG00000258667	HIF1A-AS2	6.0	7.5E-04
ENSG00000079691	LRRC16A	5.9	9.8E-04
ENSG00000184258	CDR1	5.6	3.2E-02
ENSG00000073282	TP63	5.4	2.6E-03
ENSG00000272870	LOC105377540	4.8	1.0E-03
ENSG00000134755	DSC2	4.7	5.9E-03
ENSG00000225764	P3H2-AS1	4.2	1.7E-02
ENSG00000198865	CCDC152	4.2	2.8E-02
ENSG00000120738	EGR1	4.2	9.8E-03
ENSG00000253882	LOC154761	4.0	1.3E-02
ENSG00000239445	ST3GAL6-AS1	3.9	1.7E-03
ENSG00000019549	SNAI2	3.8	3.9E-02
ENSG00000261409	N/A	3.8	3.8E-02
ENSG00000102024	PLS3	3.8	1.2E-02
ENSG00000199879	RNU1-5	3.7	4.8E-02
ENSG00000222701	RNY4P7	3.6	3.9E-02
ENSG00000249096	LOC102723766	3.4	4.3E-03
ENSG00000255693	FLJ41278	3.2	3.4E-02
ENSG00000236591	LOC105378047	3.2	4.1E-03
ENSG00000008311	AASS	3.2	1.2E-02
ENSG00000253661	ZFHX4-AS1	3.2	4.2E-02
ENSG00000228639	LOC102723505	3.1	1.3E-02
ENSG00000064225	ST3GAL6	3.1	1.9E-02
ENSG00000198780	FAM169A	3.1	3.1E-02
ENSG00000253140	LOC105375822	3.0	3.2E-02
ENSG00000023171	GRAMD1B	2.9	2.8E-02
ENSG00000256594	LOC374443	2.9	1.1E-02
ENSG00000182732	RGS6	2.9	2.2E-02
ENSG00000267121	LOC339192	2.8	1.3E-02
ENSG00000043462	LCP2	2.7	6.3E-03
ENSG00000252847	RNU2-46P	2.7	1.8E-02
ENSG00000089057	SLC23A2	2.7	4.2E-03
ENSG00000196668	LINC00173	2.7	3.4E-02
ENSG00000229671	LINC01150	2.7	1.9E-02
ENSG00000211772	TRBV3-1	2.7	3.7E-03
ENSG00000264468	MIR4520-1	2.7	4.3E-02
ENSG00000182621	PLCB1	2.7	5.0E-02
ENSG00000006659	LGALS14	2.7	4.1E-03
ENSG00000183850	ZNF730	2.7	4.3E-02
ENSG00000170379	FAM115C	2.6	2.8E-02
ENSG00000186352	ANKRD37	2.6	1.5E-02
ENSG00000090376	IRAK3	2.5	1.2E-02
ENSG00000160856	FCRL3	2.5	2.1E-02
ENSG00000257027	N/A	2.5	5.9E-03
ENSG00000210195	MT-TT	2.5	3.1E-02
ENSG00000170345	FOS	2.5	3.1E-02
ENSG00000196329	GIMAP5	2.5	1.3E-03
ENSG00000186810	CXCR3	2.5	1.9E-02
ENSG00000181074	OR52N4	2.4	2.6E-02
ENSG00000078269	SYNJ2	2.4	4.6E-02
ENSG00000247095	MIR210HG	2.4	8.8E-03
ENSG00000075213	SEMA3A	2.4	2.0E-02
ENSG00000138185	ENTPD1	2.4	4.4E-02
ENSG00000095637	SORBS1	2.4	2.7E-02
ENSG00000253047	N/A	2.4	7.6E-03
ENSG00000091656	ZFHX4	2.4	3.7E-02
ENSG00000115318	LOXL3	2.4	1.1E-02
ENSG00000150991	UBC	2.4	2.4E-03
ENSG00000137507	LRRC32	2.4	4.9E-02
ENSG00000196747	HIST1H2AG	2.4	3.4E-02
ENSG00000104419	NDRG1	2.3	3.4E-02

ENSG00000213988	ZNF253	2.3	4.8E-02
ENSG00000111674	ENO2	2.3	4.9E-03
ENSG00000200972	RNU5A	2.3	1.3E-02
ENSG00000244620	N/A	2.3	1.3E-02
ENSG00000114268	PFKFB4	2.3	5.0E-02
ENSG00000101336	HCK	2.3	7.9E-03
ENSG00000050030	KIAA2022	2.3	4.5E-02
ENSG00000180543	TSPYL5	2.3	9.7E-03
ENSG00000163564	PYHIN1	2.3	2.3E-02
ENSG00000154760	SLFN13	2.2	2.5E-02
ENSG00000084710	EFR3B	2.2	6.6E-03
ENSG00000257345	LOC105369906	2.2	4.3E-02
ENSG00000175265	GOLGA8B	2.2	3.7E-02
ENSG00000251259	N/A	2.2	4.6E-02
ENSG00000245694	CRNDE	2.2	2.8E-02
ENSG00000255733	IFNG-AS1	2.2	1.3E-02
ENSG00000252026	RNU6-1262P	2.2	4.7E-02
ENSG00000232445	LOC101927746	2.2	2.4E-02
ENSG00000196968	FUT11	2.2	3.2E-03
ENSG00000161249	DMKN	2.2	1.7E-02
ENSG00000173933	RBM4	2.2	3.4E-02
ENSG00000101311	FERMT1	2.1	4.6E-02
ENSG00000273727	N/A	2.1	6.8E-03
ENSG00000059804	SLC2A3	2.1	7.7E-03
ENSG00000075826	SEC31B	2.1	1.8E-02
ENSG00000198089	SFI1	2.1	2.4E-02
ENSG00000202408	RNU1-5	2.1	5.0E-02
ENSG00000138795	LEF1	2.1	2.3E-02
ENSG00000235823	OLMALINC	2.1	2.6E-02
ENSG00000133328	HRASLS2	2.1	3.0E-02
ENSG00000179144	GIMAP7	2.1	4.2E-02
ENSG00000152256	PDK1	2.1	1.6E-03
ENSG00000277258	PCGF2	2.1	9.1E-03
ENSG00000007944	MYLIP	2.1	3.2E-02
ENSG00000236352	N/A	2.0	4.0E-02
ENSG00000153930	ANKFN1	2.0	4.2E-02
ENSG00000167766	ZNF83	2.0	2.5E-02

Table S1. Significantly up-regulated transcripts identified from microarray analysis following miR-6891 inhibition (HSA miR-6891-5p inhibition vs. control samples). Identified genes are putative, direct targets of HSA-miR-6891-5p. High confidence putative targets are shown in red and additionally contain a predicted HSA-miR-6891-5p binding site within the 3' UTR of the indicated gene as identified by in silico miRNA target prediction.

Ensemble Gene ID	Gene Symbol	Fold Change	FDR
ENSG00000131016	AKAP12	-5.7	2.7E-02
ENSG00000140450	ARRDC4	-5.5	6.3E-04
ENSG00000253874	IGLVIV-66-1	-4.3	4.0E-02
ENSG00000104722	NEFM	-4.2	3.5E-04
ENSG00000174827	PDZK1	-4.2	4.2E-02
ENSG00000156689	GLYATL2	-4.1	2.5E-02
ENSG00000170100	ZNF778	-4.0	2.0E-02
ENSG00000169245	CXCL10	-3.9	1.8E-02
ENSG00000269586	CT45A10	-3.8	9.4E-03
ENSG00000278705	HIST1H4B	-3.8	3.0E-02
ENSG00000005249	PRKAR2B	-3.7	3.0E-02
ENSG00000117009	KMO	-3.6	1.5E-02
ENSG00000130956	HABP4	-3.5	8.2E-03
ENSG00000135052	GOLM1	-3.3	2.1E-03
ENSG00000211676	IGLJ2	-3.3	1.1E-02
ENSG00000138760	SCARB2	-3.2	1.7E-02
ENSG00000249049	N/A	-3.2	2.0E-02
ENSG00000226562	CYP4F26P	-3.1	4.8E-02
ENSG00000126010	GRPR	-3.1	5.8E-03
ENSG00000103942	HOMER2	-3.0	2.1E-02
ENSG00000273118	LOC105373862	-3.0	4.5E-02
ENSG00000131470	PSMC3IP	-3.0	3.1E-03
ENSG00000137941	TTLL7	-3.0	2.2E-02
ENSG00000137942	FNBP1L	-2.9	2.4E-02
ENSG00000238648	TRI-TAT2-3	-2.9	3.7E-02
ENSG00000211648	IGLV1-47	-2.9	4.2E-02
ENSG00000165912	PACSIN3	-2.9	1.1E-03
ENSG00000166342	NETO1	-2.8	6.2E-03
ENSG00000102241	HTATSF1	-2.8	7.1E-03
ENSG00000263961	C1ORF186	-2.8	4.7E-03
ENSG00000154059	IMPACT	-2.8	8.5E-03
ENSG00000158869	FCER1G	-2.8	3.9E-02
ENSG00000170500	LONRF2	-2.8	2.5E-02
ENSG00000132970	WASF3	-2.7	3.1E-02
ENSG00000111537	IFNG	-2.7	2.6E-02
ENSG00000277586	NEFL	-2.7	4.8E-03
ENSG00000140465	CYP1A1	-2.7	2.3E-02
ENSG00000238244	GABARAPL3	-2.7	3.3E-02
ENSG00000087191	PSMC5	-2.7	7.5E-03
ENSG00000135698	MPHOSPH6	-2.7	5.0E-03
ENSG00000137267	TUBB2A	-2.7	3.3E-02
ENSG00000000003	TSPAN6	-2.6	2.5E-02
ENSG00000132465	IGJ	-2.6	5.7E-03
ENSG00000072133	RPS6KA6	-2.5	3.5E-02
ENSG00000100557	C14ORF105	-2.5	2.5E-03
ENSG00000170846	LOC93622	-2.5	2.7E-03
ENSG00000214941	ZSWIM7	-2.5	2.2E-02
ENSG00000169957	ZNF768	-2.5	1.5E-02
ENSG00000088256	GNA11	-2.5	9.8E-03
ENSG00000136634	IL10	-2.5	8.8E-03
ENSG00000110090	CPT1A	-2.5	1.1E-02
ENSG00000173212	C1ORF161	-2.4	1.8E-02
ENSG00000100292	HMOX1	-2.4	3.9E-03
ENSG00000148468	FAM171A1	-2.4	4.0E-02
ENSG00000169857	AVEN	-2.4	2.7E-02
ENSG00000198648	STK39	-2.4	2.3E-02
ENSG00000082458	DLG3	-2.4	3.1E-02
ENSG00000163823	CCR1	-2.4	3.1E-02
ENSG00000137673	MMP7	-2.4	1.5E-02
ENSG00000182534	MXRA7	-2.3	4.4E-02
ENSG00000198283	OR5B21	-2.3	3.8E-02
ENSG00000185900	SGK196	-2.3	1.9E-03
ENSG00000138678	AGPAT9	-2.3	1.2E-02
ENSG00000132170	PPARG	-2.3	2.3E-02
ENSG00000108830	RND2	-2.3	2.1E-02
ENSG00000175857	GAPT	-2.3	3.6E-02
ENSG00000198729	PPP1R14C	-2.3	4.1E-02

ENSG00000213886	UBD	-2.3	2.9E-02
ENSG00000125869	C20ORF103	-2.3	1.1E-02
ENSG00000183723	CMTM4	-2.2	3.0E-02
ENSG00000162627	SNX7	-2.2	1.9E-02
ENSG00000169914	OTUD3	-2.2	4.3E-02
ENSG00000179010	MRFAP1	-2.2	1.3E-02
ENSG00000167674	HDGFRP2	-2.2	7.4E-03
ENSG00000021355	SERPINB1	-2.2	9.8E-03
ENSG00000181191	PJA1	-2.2	1.4E-02
ENSG00000188558	OR2G6	-2.2	1.8E-02
ENSG00000115109	EPB41L5	-2.2	4.0E-02
ENSG00000129250	KIF1C	-2.2	1.8E-03
ENSG00000117174	ZNHIT6	-2.2	2.4E-02
ENSG00000211972	IGHV3-66	-2.1	4.0E-02
ENSG00000235493	N/A	-2.1	3.6E-02
ENSG00000203661	OR2T5	-2.1	4.4E-02
ENSG00000183624	C3ORF37	-2.1	8.4E-03
ENSG00000114446	IFT57	-2.1	2.3E-03
ENSG00000180015	LOC285442	-2.1	4.3E-02
ENSG00000169435	RASSF6	-2.1	6.9E-03
ENSG00000111275	ALDH2	-2.1	2.3E-02
ENSG00000169750	RAC3	-2.1	4.1E-03
ENSG00000102390	CXORF26	-2.1	1.9E-02
ENSG00000183688	FAM101B	-2.0	1.2E-02
ENSG00000260943	LOC101930164	-2.0	3.1E-02
ENSG00000176903	PNMA1	-2.0	2.3E-02
ENSG00000067533	RRP15	-2.0	2.1E-02
ENSG00000160633	SAFB	-2.0	3.0E-03
ENSG00000101096	NFATC2	-2.0	3.4E-02
ENSG00000211677	IGLV2-11	-2.0	1.1E-02
ENSG00000096717	SIRT1	-2.0	2.7E-02
ENSG00000188641	DPYD	-2.0	2.8E-02

Table S2. Significantly down-regulated transcripts identified from microarray analysis (miR-6891-5p inhibition vs. control samples). Identified mRNA transcripts presumably reflect indirect effects of miR-6891-5p inhibition on lymphoblastoid cells rather than direct miRNA binding of listed transcripts.

FUNCTIONAL ENRICHMENT OF SIGNIFICANT UPREGULATED TRANSCRIPTS (Inhibition vs. Control)		
**Gene Ontology Term**	**P-Value**	**Fold Enrichment**
mesoderm development	1.5E-03	1.7E+01
ectoderm and mesoderm interaction	6.2E-03	3.1E+02
myeloid leukocyte activation	8.9E-03	2.1E+01
cell activation	1.2E-02	5.5E+00
positive regulation of macromolecule biosynthetic process	1.5E-02	3.4E+00
positive regulation of cellular biosynthetic process	1.8E-02	3.2E+00
positive regulation of biosynthetic process	1.9E-02	3.2E+00
response to organic substance	2.3E-02	3.1E+00
mast cell activation	2.5E-02	7.9E+01
positive regulation of transcription from RNA polymerase II promoter	2.7E-02	4.2E+00
positive regulation of transcription	3.0E-02	3.3E+00
monosaccharide metabolic process	3.1E-02	5.7E+00
positive regulation of gene expression	3.3E-02	3.2E+00
leukocyte activation	3.9E-02	5.2E+00
positive regulation of nucleobase, nucleoside, nucleotide and nucleic acid metabolic process	4.3E-02	3.0E+00
learning or memory	4.6E-02	8.5E+00
positive regulation of macromolecule metabolic process	4.7E-02	2.6E+00
positive regulation of nitrogen compound metabolic process	4.8E-02	2.9E+00
negative regulation of transcription from RNA polymerase II promoter	4.9E-02	4.7E+00

FUNCTIONAL ENRICHMENT OF SIGNIFICANT DOWNREGULATED TRANSCRIPTS (Inhibition vs. Control)		
**Gene Ontology Term**	**P-Value**	**Fold Enrichment**
regulation of production of molecular mediator of immune response	8.6E-04	2.1E+01
regulation of membrane protein ectodomain proteolysis	1.6E-03	5.0E+01
regulation of leukocyte mediated immunity	2.7E-03	1.4E+01
regulation of cytokine production during immune response	3.0E-03	3.6E+01
heterocycle catabolic process	5.3E-03	1.1E+01
regulation of B cell mediated immunity	5.3E-03	2.7E+01
regulation of immunoglobulin mediated immune response	5.3E-03	2.7E+01
behavior	5.5E-03	3.7E+00
taxis	6.2E-03	6.7E+00
chemotaxis	6.2E-03	6.7E+00
regulation of cytokine production	9.5E-03	5.9E+00
regulation of immune effector process	1.1E-02	8.5E+00
negative regulation of cytokine production	1.3E-02	1.7E+01
response to organic cyclic substance	1.8E-02	7.1E+00

receptor biosynthetic process	1.8E-02	1.1E+02
regulation of mast cell cytokine production	1.8E-02	1.1E+02
positive regulation of MHC class II biosynthetic process	2.3E-02	8.6E+01
regulation of gene-specific transcription	2.4E-02	6.4E+00
regulation of protein catabolic process	2.4E-02	1.2E+01
regulation of proteolysis	2.5E-02	1.2E+01
organic ether metabolic process	2.5E-02	1.2E+01
regulation of lymphocyte mediated immunity	2.5E-02	1.2E+01
response to alkaloid	2.5E-02	1.2E+01
regulation of adaptive immune response based on somatic recombination of immune receptors built from immunoglobulin superfamily domains	2.6E-02	1.2E+01
regulation of cell proliferation	2.7E-02	2.5E+00
regulation of cellular localization	2.7E-02	4.3E+00
regulation of adaptive immune response	2.7E-02	1.2E+01
positive regulation of chemokine biosynthetic process	3.2E-02	6.1E+01
white fat cell differentiation	3.2E-02	6.1E+01
regulation of cellular catabolic process	3.2E-02	1.1E+01
regulation of MHC class II biosynthetic process	3.6E-02	5.4E+01
response to hyperoxia	3.6E-02	5.4E+01
locomotory behavior	3.7E-02	3.9E+00
immune response	3.8E-02	2.5E+00
negative regulation of multicellular organismal process	3.9E-02	5.2E+00
positive regulation of membrane protein ectodomain proteolysis	4.1E-02	4.8E+01
regulation of chemokine biosynthetic process	4.5E-02	4.3E+01
regulation of myeloid leukocyte mediated immunity	4.5E-02	4.3E+01
regulation of cytokine biosynthetic process	4.5E-02	8.7E+00
regulation of inflammatory response	4.7E-02	8.5E+00

Table S3. Gene ontology (GO) functional enrichment of significant, differentially expressed transcripts identified from microarray analysis. GO biological processes (level 4) were annotated using a p value cutoff of 0.05.

Sample ID	Family	Relationship	Disease Status	HLA-A	HLA-B	HLA-C	HLA-DRB1	HLA-DQA1	HLA-DQB1	HLA-DPA1	HLA-DPB1
ID57	1	Father	Affected	01:01:01	08:01:01	07:01:01	03:01:01	05:01:01	02:01:01	02:01:02	01:01:01
		Father		02:01:01	40:02:01	15:02:01	04:01:01	03:03:01	03:01:01	01:03:01	04:02:01
ID58	1	Daughter	Unaffected	01:01:01	08:01:01	07:01:01	03:01:01	05:01:01	02:01:01	02:01:02	01:01:01
		Daughter		29:02:01	44:03:01	16:01:01	07:01:01	02:01:01	02:02:01	02:02:02	01:01:01
ID38	1	Daughter	Affected	01:01:01	08:01:01	07:01:01	03:01:01	05:01:01	02:01:01	02:01:02	01:01:01
		Dauthrer		02:01:01	40:01:02	03:04:01	13:01:01	01:03:01	06:03:01	02:01:01	02:01:02
ID37	2	Mother	Unaffected	26:01:01	38:01:01	12:03:01	04:02:01	03:01:01	03:02:01	01:03:01	04:01:01
		Mother		24:02:01	08:01:01	07:01:01	03:01:01	05:01:01	02:01:01	01:03:01	04:01:01
ID36	2	Son	Affected	26:01:01	38:01:01	12:03:01	04:02:01	03:01:01	03:02:01	01:03:01	04:01:01
		Son		32:01:01	07:05:01	04:01:01	10:01:01	01:05:01	05:01:01	01:03:01	04:01:01
ID18	3	N/A	Affected	01:01:01	08:01:01	07:01:01	03:01:01	05:01:01	02:01:01	01:03:01	04:01:01
		N/A		02:01:01	35:01:01	04:01:01	04:02:01	03:01:01	03:02:01	01:03:01	04:01:01

Table S4. High resolution HLA genotyping results of B-LCLs obtained from patients with selective IgA deficiency and unaffected, related family members. All samples were obtained from the Coriell Biorepository. Phased MHC haplotypes were inferred from related individuals when available (ID57, ID58, ID38, ID37 and ID36) and based upon common MHC haplotypes otherwise (ID18).
